# Low physical activity, high television viewing and poor sleep duration cluster in overweight and obese adults; a cross-sectional study of 398,984 participants from the UK Biobank

**DOI:** 10.1186/s12966-017-0514-y

**Published:** 2017-04-28

**Authors:** Sophie Cassidy, Josephine Y. Chau, Michael Catt, Adrian Bauman, Michael I. Trenell

**Affiliations:** 1 Institute of Cellular Medicine, Faculty of Medical Sciences, Newcastle University, Newcastle upon Tyne, NE2 4HH UK; 2 Prevention Research Collaboration, Sydney School of Public Health, Charles Perkins Centre D17, Level 6 The Hub, University of Sydney, Sydney, NSW 2006 Australia; 3 Institute of Neuroscience, Faculty of Medical Sciences, Newcastle University, Newcastle upon Tyne, NE2 4HH UK

**Keywords:** Physical activity, Sedentary, Sleep, Lifestyle, Obesity

## Abstract

**Background:**

An unhealthy lifestyle is one of the greatest contributors to obesity. A number of behaviours are linked with obesity, but are often measured separately. The UK Biobank cohort of >500,000 participants allows us to explore these behaviours simultaneously. We therefore aimed to compare physical activity, television (TV) viewing and sleep duration across body mass index (BMI) categories in a large sample of UK adults.

**Methods:**

UK Biobank participants were recruited and baseline measures were taken between 2007 and 2010 and data analysis was performed in 2015. BMI was measured objectively using trained staff. Self-report questionnaires were used to measure lifestyle behaviours including the international physical activity questionnaire (IPAQ-short form) for physical activity. During data analysis, six groups were defined based on BMI; ‘Underweight’ (*n* = 2026), ‘Normal weight’ (*n* = 132,372), ‘Overweight (*n* = 171,030), ‘Obese I’ (*n* = 67,903), ‘Obese II’ (*n* = 18,653) and ‘Obese III’ (*n* = 7000). The odds of reporting unhealthy lifestyle behaviours (low physical activity, high TV viewing or poor sleep duration) were compared across BMI groups using logistic regression analysis.

**Results:**

Overweight and obese adults were more likely to report low levels of physical activity (≤967.5 MET.mins/wk) (‘Overweight’-OR [95% CI]: 1.23 [1.20 to 1.26], ‘Obese I’ 1.66 [1.61–1.71], ‘Obese II’ 2.21 [2.12–2.30], and ‘Obese III’ 3.13 [2.95 to 3.23]) compared to ‘Normal weight’ adults. The odds of reporting high TV viewing (3 h/day) was greater in ‘Overweight’ (1.52 [1.48 to 1.55]) and obese adults (‘Obese I’ 2.06 [2.00–2.12], ‘Obese II’ 2.69 [2.58–2.80], ‘Obese III’ 3.26 [3.07 to 3.47]), and poor sleep duration (<7, >8 h/night) was higher in ‘Overweight’ (1.09 [1.07 to 1.12]) and obese adults (‘Obese I’ 1.31 [1.27–1.34], ‘Obese II’ 1.50 [1.44–1.56], ‘Obese III’ (1.78 [1.68 to 1.89]) compared to the ‘Normal weight’ group. These lifestyle behaviours were clustered, the odds of reporting simultaneous low physical activity, high TV viewing and poor sleep (unhealthy behavioural phenotype) was higher than reporting these behaviours independently, in overweight and obese groups. ‘Obese III’ adults were almost six times more likely (5.47 [4.96 to 6.05]) to report an unhealthy behavioural phenotype compared to the ‘Normal weight’ group.

**Conclusions:**

Overweight and obese adults report low levels of physical activity, high TV viewing and poor sleep duration. These behaviours seem to cluster and collectively expose individuals to greater risk of obesity. Multiple lifestyle behaviours should be targeted in future interventions.

**Electronic supplementary material:**

The online version of this article (doi:10.1186/s12966-017-0514-y) contains supplementary material, which is available to authorized users.

## Background

Globally, the proportion of adults with a normal body mass index (BMI) is reducing [1, 2] and prediction models indicate that this shift in BMI will continue, so that by 2030 the number of obese adults will have risen by 11 million in the UK alone [3]. Fifty years ago there was no uniformity when measuring obesity, [4] yet the adoption of BMI recommended standards by the World Health Organisation (WHO) [5] meant a standardised definition was created for national surveillance, making it an effective measure for population wide comparisons.

Global and UK strategies for obesity prevention and management promote lifestyle modification, including increased physical activity and healthy nutritional intake, and emphasise their importance before any pharmacological intervention [6–8]. Physical activity is inversely associated with obesity, [9] and improvements in activity levels improve fat oxidation [10] and other determinants of obesity [11]. Across the energy expenditure spectrum, and within a 24 h period, sedentary behaviour and sleep also influence a person’s metabolism, [12, 13] and have both been linked to obesity. The direction of association between obesity and sedentary behaviour is not certain, and it remains unclear whether obesity is a cause or consequence of total daily sitting time [14–18]. There is more evidence for television (TV) sitting time and obesity, however other unhealthy behaviours such as snacking are related to TV viewing. Additionally, many obese individuals suffer from sleep apnoea, [19] yet even when controlling for this condition, they have a higher prevalence of short sleep. [20] The strong association between short sleep and increased BMI is well documented, [21] and has been attributed to hormonal imbalances and reductions in energy expenditure [13].

Energy intake is well established as a risk factor for obesity, and diet recommendations form a major part of national guidelines for the prevention and management of weight gain [6]. In this study, energy expenditure was the main focus. As physical activity, sedentary behaviour and sleep are synergistically related to energy expenditure, clustering of these lifestyle behaviours in obesity is expected. Despite this, current policies and interventions to tackle the growing obesity trend often overlook multiple risk behaviours [22]. The UK Biobank provides us with a novel opportunity to simultaneously assess these lifestyle behaviours in a population based sample of UK adults. The UK Biobank is a population-based cohort of 502,664 adults aged 37–73 years old, recruited and assessed between 2007 and 2010 [23]. Our aim was to measure physical activity, TV viewing and sleep duration across BMI categories, and to explore clustering between these lifestyle behaviours.

## Methods

### Population and study design

**Fig. 1:**
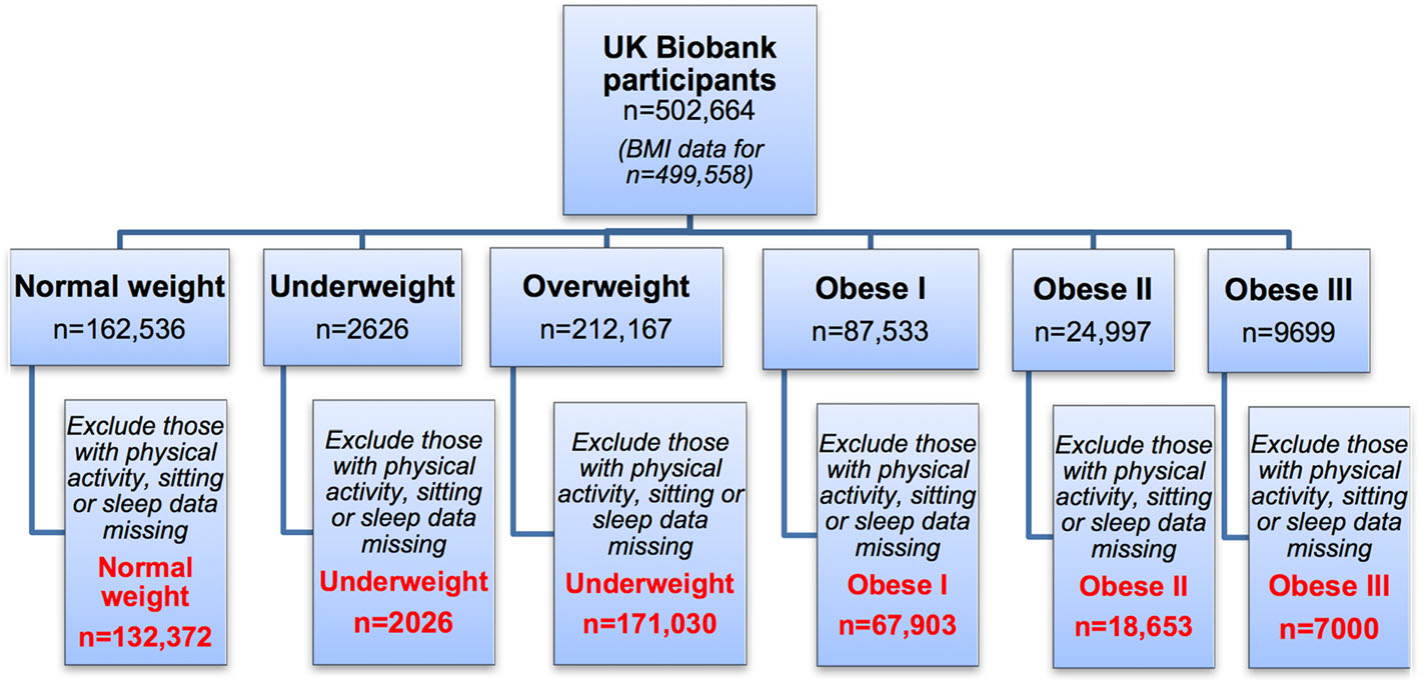
Flow chart to show how BMI groups were defined (final 4 groups shown in *red*)

A cross sectional analysis was conducted on baseline data from the UK Biobank in 2015. Only individuals who had data on physical activity, TV viewing and sleep were included in this analysis (*n* = 398,984) (Fig. 1). Details of UK Biobank methods and procedures have been previously published [23]. All data extracted were de-identified for analysis.

### Baseline measurements

During a verbal interview, disease status was entered and verified by a UK Biobank nurse whereas information on lifestyle behaviours were collected from the touchscreen questionnaire. Physical activity was assessed using six items in the validated Short International Physical Activity Questionnaire (IPAQ) [24]. Time spent in vigorous, moderate, and walking activity was weighted by the energy expended for these categories of activity to produce MET.mins/week of physical activity, which is referred to as ‘total physical activity’. Data processing rules published by IPAQ were followed [25].

TV viewing [26] was used as a marker of sedentary behaviour. Participants were asked; “In a typical day, how many hours do you spend watching television?” based on previous literature [18]. This was asked twice to those who responded >8 h, therefore high values were deemed robust. To measure sleep duration, participants were asked “About how many hours sleep do you get in every 24 h? (please include naps)". This was asked twice to those who responded >12 h.

For the other lifestyle behaviours, the touchscreen questionnaire summarised the current/past smoking and alcohol status of the participant and diet intake was reported using the Food Frequency Questionnaire (FFQ) [27]. A short subset of FFQ questions which provided information of commonly eaten food groups and common sources of various nutrients were selected for use in the UK biobank (see [23] for more information). Information on fresh/dried fruit, salad and cooked/raw vegetables were combined to create a binary variable to identify individuals who did and did not meet the UK’s current guidelines on fruit and vegetable consumption (five per day) [28]. Participants were asked ‘have you made any major changes to your diet in the last 5 years?’ and were also required to select any of the following foods they ‘NEVER eat’; eggs, dairy, wheat or sugar.

BMI was calculated from: weight(kg)/height(m).^2^ Weight was measured using the Tanita BC-418MA body composition analyser, to the nearest 0.1 kg and height was measured using a Seca 202 height measure. Bioimpedence (Tanita BC-418MA) was used to measure body fat. Trained staff took these measures and participants were required to remove shoes and heavy outer clothing. Waist circumference was measured at the level of the umbilicus using a Wessex non-stretchable sprung tape measure, which has previously been adopted in large health studies [29]. Participants were asked to adjust clothing for an accurate measure, and all staff were trained in taking these measures.

### Data analysis

BMI groups were defined based on WHO recommended cut points [30] which are; <18.5 kg/m^2^ (underweight), 18.5–24.99 kg/m^2^ (normal weight), 25–29.99 kg/m^2^ (overweight), 30–34.99 kg/m^2^ (obese I), 35–39.99 kg/m^2^ (Obese II) and ≥40 kg/m^2^ (Obese III). Individuals with missing data on either total physical activity, sitting time or sleep were excluded (*n* = 100,574). Additional file 1 shows the socio-demographics of missing cases, which were similar to the main cohort, but there was a greater proportion of obese individuals aged 60–70 years and there was a lower proportion of males across all groups.

Total physical activity, vigorous, moderate and walking mins and TV viewing time were categorised into 4 groups. These groups were based on the quartile demarcators for the ‘Normal weight’ BMI adults, within each variable. Total physical activity groups were labelled as ‘low physical activity’ (lowest quartile: ≤967.5 MET.mins/wk) and ‘high physical activity’ (highest quartile: >3786 MET.mins/wk) and TV viewing was labelled as ‘low TV viewing’ (lowest quartile: ≤1 hour/day) and ‘high TV viewing’ (highest quartile: >3 hour/day). As the relationship between obesity and sleep duration is not a linear, sleep duration was split using pre-defined thresholds (<7, 7–8, >8 h/night) from the literature [20, 31]. Sleep duration was labelled as ‘poor sleep’ (<7 or >8 h/night) and ‘good sleep’ (7–8 h/night).

### Statistical analysis

All data analyses were performed using SPSS, version 21.0 (IBM, Armonk, NY, USA). Due to the large sample size, Pearson’s chi squared deemed any small difference in group proportions as significant, therefore these results are not reported. Physical activity, TV viewing and sleep duration were statistically analysed across BMI groups. Binary logistic regression was used to determine the odds of reporting low physical activity, high TV viewing and poor sleep duration separately, according to BMI group. We also looked at the clustering of these behaviours. Participants were categorised as having an ‘unhealthy phenotype’ if they were categorised in all of the following groups; low total physical activity, high TV viewing and poor sleep duration. As BMI isn’t a direct measure of obesity, the National Institute for Health and Care Excellence (NICE) recommended combining BMI in conjunction with waist circumference [1]. Due to the spread of waist circumference in ‘overweight’ and ‘obese I’ groups, we further classified these groups by waist circumference and performed the above analysis (see Additional file 1). Adjusted odds ratios, with 95% confidence intervals were reported. All logistic regression models were adjusted for: age (reference=”40–50”), gender (reference=”Female”), Townsend Deprivation Index (reference=”least deprived”), Ethnicity (reference=”White/British”), Alcohol (reference=”never”), Smoking (reference=”Never”), Meets fruit/veg guidelines (reference=”Yes”), Sleep Apnoea (reference=”No”), Cardio-metabolic disease (reference=”No”). Cardio-metabolic disease and sleep apnoea were identified as confounders because cardio-metabolic disease and BMI are strongly associated, and obesity results in sleep apnoea which disturbs sleep. Of the 398,984 cohort, data was missing for; Townsend Deprivation Index (0.1%), Ethnicity (0. 3%), and fruit and vegetable guidelines (0.015%) therefore these cases were excluded from the logistic regression models.

## Results

Of the 398,984 UK Biobank participants who had data on physical activity, TV viewing and sleep; 33% (*n* = 132,372) were ‘normal weight’, 0.5% (*n* = 2026) ‘Underweight’, 43% (*n* = 171,030) ‘Overweight’, 17% (*n* = 67,903) ‘Obese I’, 5% (*n* = 18,653) ‘Obese II’, and 2% (*n* = 7000) ‘Obese III’ (Fig. 1). Table 1 displays the socio-demographics of BMI groups.

**Table 1:** Socio-demographics, anthropometry and disease status within each BMI group (*n* = 398,984)

	% Within each disease group					
	Under weight <18.5 (*n* = 2026)	Normal weight 18.5–24.99 (*n* = 132,372)	Over weight 25–29.99 (*n* = 171,030)	Obese I 30–34.99 (*n* = 67,903)	Obese II 35–39.99 (*n* = 18,653)	Obese III ≥40 (*n* = 7000)
% Male	20.9	36.3	55.2	54.1	43.2	34.3
Age *(n)*	*2026*	*132,372*	*171,030*	*67,903*	*18,653*	*7000*
40–49	27.7	28.1	22.6	21.6	23.1	26.9
50–59	36.6	33.7	32.4	32.2	36.0	39.2
60–70	35.7	38.2	45.1	44.3	40.9	33.9
Tanita body fat % (mean ± SD)	18.6 ± 5.4	26.7 ± 7.1	30.8 ± 7.3	36.0 ± 7.2	41.3 ± 6.8	46.3 ± 6.2
Waist circumference groups (MALES) *(n)*	*424*	*48,049*	*94,322*	*36,730*	*8060*	*2407*
<94 cm (low risk)	99.8	90.1	35.6	1.8	0.0	0.1
94–102 cm (high risk)	0.2	9.7	49.9	24.8	1.3	0.1
>102 cm (very high risk)	0.0	0.2	14.5	73.4	98.7	99.8
Waist circumference groups (FEMALES) *(n)*	*1602*	*84,302*	*76,688*	*31,151*	*10,590*	*4581*
<80 cm (low risk)	99.5	78.6	20.2	0.8	0.0	0.0
80–88 cm (high risk)	0.2	19.4	48.7	14.2	1.1	0.1
>88 cm (very high risk)	0.2	2.0	31.1	85.0	98.8	99.9
Cardio-metabolic disease	35.8	37.2	56.1	71.8	81.0	86.8
Sleep apnoea (n)	0.0 (1)	0.1 (76)	0.2 (345)	0.6 (404)	1.4 (267)	2.7 (188)
Townsend deprivation quintile *(n)*	*2023*	*132,214*	*170,825*	*67,811*	*18,622*	*6986*
1 (least deprived)	17.4	22.0	21.4	18.6	15.0	12.0
2	17.3	20.9	21.0	19.3	17.2	14.0
3	18.1	20.2	20.5	19.8	18.9	18.0
4	20.8	19.6	19.7	20.7	21.9	22.4
5 (most deprived)	26.4	17.3	17.4	21.7	27.0	33.7
Ethnicity *(n)*	*2016*	*132,020*	*170,546*	*67,672*	*18,594*	*6970*
White/British	93.5	95.3	95.0	94.5	94.2	93.3
Mixed	1.0	0.6	0.5	0.6	0.6	0.8
Asian	2.3	1.8	1.9	1.7	1.3	1.1
Black African	0.4	0.8	1.5	2.2	2.9	3.7
Chinese	1.2	0.6	0.2	0.1	0.0	0.0
Other	1.5	0.8	0.8	0.9	1.0	1.0

**Fig. 2:**
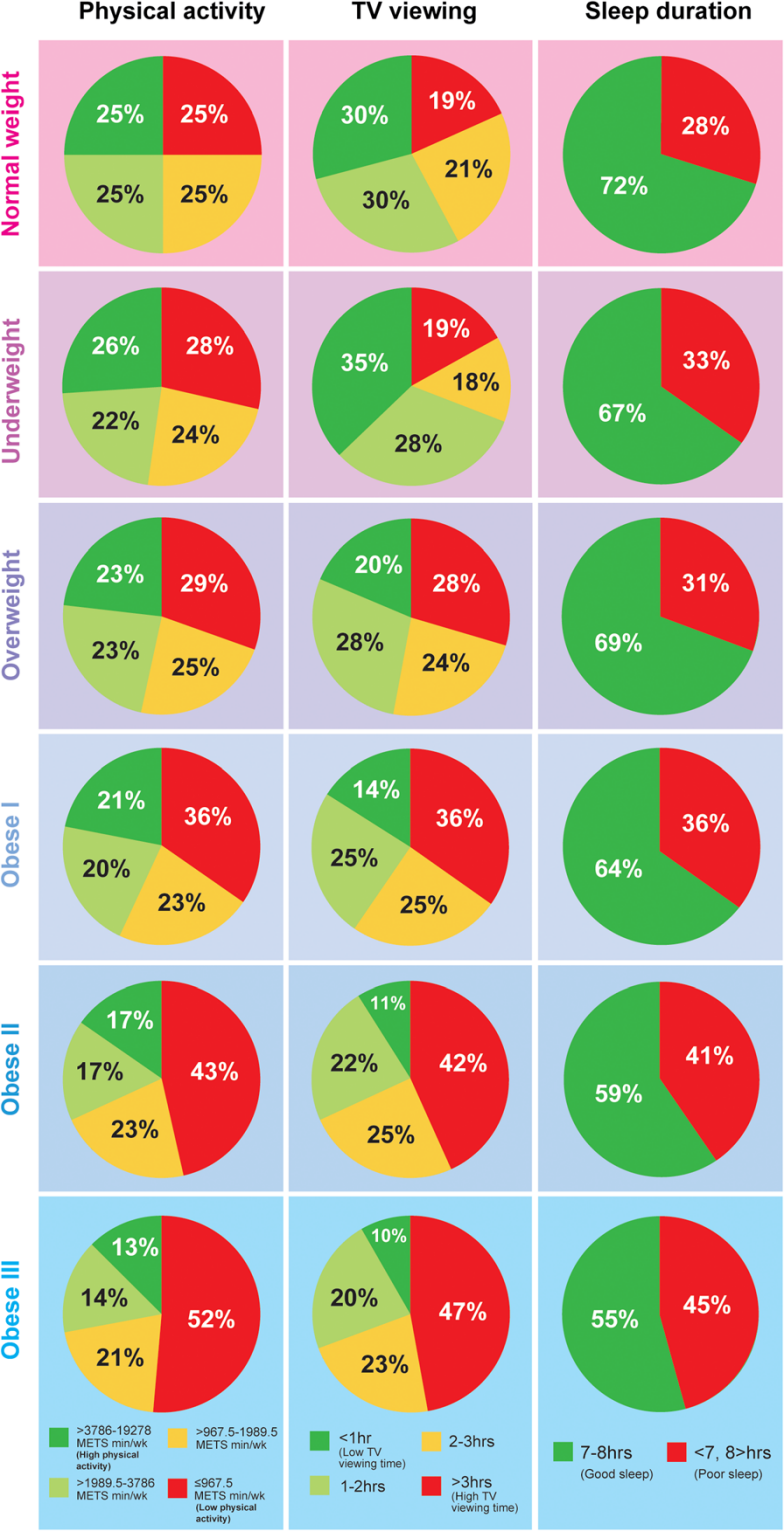
Distribution of physical activity, TV viewing and sleep duration across BMI groups. Visual representation of lifestyle behaviours reported in Table 2. Red indicates unhealthy and green indicated healthy lifestyle behaviours

Total weekly physical activity decreased across BMI groups with 25% of ‘Normal weight’ adults reaching the high quartile of physical activity (>3786 METs.min.wk) compared to 12.7% of ‘Obese III’ adults (Table 2). Fifteen per cent of ‘Normal weight’ adults did not meet the UK’s physical activity recommendations, which rose across BMI groups to 38.2% in ‘Obese III’ adults. The proportion of adults reporting high TV viewing increased across BMI groups (‘Normal weight’, 19.2% *vs.* ‘Overweight’, 28.3% *vs.* ‘Obese III’, 47.1%) so that almost half of ‘Obese III’ adults reported TV viewing for greater than >3 h per day (Table 2). Good sleep duration declined across BMI groups with 72% of ‘Normal weight’ adults reporting 7–8 h sleep per night and only 54.5% of ‘Obese III’ adults reporting similar levels (Table 2). Figure 2 is a visual representation of the differences in these lifestyle behaviours across BMI groups.

**Table 2:** Lifestyle characteristics including physical activity, TV viewing, sleep, smoking, alcohol and diet, within each BMI group (*n* = 398,984)

	% Within each disease group					
	Under weight <18.5 (*n* = 2026)	Normal weight 18.5–24.99 (*n* = 132,372)	Over weight 25–29.99 (*n* = 171,030)	Obese I 30–34.99 (*n* = 67,903)	Obese II 35–39.99 (*n* = 18,653)	Obese III ≥40 (*n* = 7000)
Physical Activity						
Total Physical activity^a^ (MET.mins/wk)	*2026*	*132,372*	*171,030*	*67,903*	*18,653*	*7000*
≤967.5 (Low physical activity)	28.0	25.0	28.9	35.6	42.9	52.5
>967.5–1989.5	23.9	25.0	24.8	23.4	22.8	20.7
>1989.5–3786	22.2	25.0	22.9	20.1	17.4	14.2
>3786 (High physical activity)	25.9	25.0	23.4	20.8	16.9	12.7
Walking^a^ (mins/day)	*2026*	*132,372*	*171,030*	*67,903*	*18,653*	*7000*
0–20	29.2	30.6	32.4	35.9	40.4	48.0
21–30	19.9	21.1	20.8	19.8	19.3	18.1
31–60	27.9	27.4	26.2	24.3	22.3	20.0
61–180	23.0	21.0	20.6	20.0	18.0	13.9
Moderate activity^a^ (mins/day)	*2026*	*132,372*	*171,030*	*67,903*	*18,653*	*7000*
0–15	25.2	20.1	23.0	28.4	33.9	42.1
16–30	30.3	33.4	32.6	31.1	31.1	28.7
31–60	23.6	25.9	24.2	21.8	19.1	17.1
61–180	20.9	20.6	20.2	18.7	15.8	12.2
Vigorous activity^a^ (mins/day)	*2026*	*132,372*	*171,030*	*67,903*	*18,653*	*7000*
0	48.6	35.5	39.5	47.6	56.2	64.0
1–20	17.9	20.8	20.4	19.2	17.4	15.0
21–45	16.9	21.1	19.2	16.2	13.3	11.6
46–180	16.5	22.6	20.8	17.0	13.2	9.4
Meets UK government physical activity guidelines^b^	*2026*	*132,372*	*171,030*	*67,903*	*18,653*	*7000*
NO	16.4	14.6	17.8	23.1	29.2	38.2
TV viewing						
TV viewing^a^ (h/day)	*2026*	*132,372*	*171,030*	*67,903*	*18,653*	*7000*
≤1 (Low TV viewing)	35.0	30.0	19.6	14.1	11.2	9.9
>1–2	28.3	29.9	27.6	24.5	21.7	19.6
>2–3	17.6	20.9	24.5	25.3	24.6	23.4
>3 (High TV viewing)	19.1	19.2	28.3	36.1	42.5	47.1
Sleep						
Sleep duration^c^ (h/night)	*2026*	*132,372*	*171,030*	*67,903*	*18,653*	*7000*
<7,>8 (Poor sleep)	33.1	28.1	30.9	36.2	40.8	45.4
7–8 (Good sleep)	66.9	72.0	69.1	63.8	59.3	54.5
Behavioural Phenotype						
Unhealthy (low physical activity, high TV viewing and poor sleep duration)	2.7% (*n* = 72)	1.6% (*n* = 2639)	2.6% (*n* = 5486)	4.4% (*n* = 3880)	6.6% (*n* = 1644)	9.9% (*n* = 956)
Other Lifestyle Behaviours						
Alcohol	*2026*	*132,372*	*171,030*	*67,903*	*18,653*	*7000*
Never	7.7	3.7	3.6	4.6	5.7	7.7
Previous	6.8	3.1	3.1	3.9	5.0	7.5
Current	85.3	93.1	93.2	91.4	89.2	84.7
Smoking	*2026*	*132,372*	*171,030*	*67,903*	*18,653*	*7000*
Never	57.3	59.2	53.3	50.3	50.5	52.5
Previous	20.9	29.8	36.4	39.7	39.8	37.9
Current	21.6	10.7	10.0	9.7	9.2	9.2
Diet						
Dietary change in past 5 years	*2023*	*132,261*	*170,862*	*67,799*	*18,600*	*6973*
YES	29.3	32.1	39.0	46.9	54.5	59.5
Meets fruit/veg guidelines	*2001*	*130,905*	*168,617*	*66,812*	*18,302*	*6887*
YES	32.7	32.1	30.4	30.3	31.7	32.3
“Never eat”	*2024*	*132,151*	*170,699*	*67,757*	*18,602*	*6970*
Never eat sugar or foods/drinks containing sugar	13.1	15.5	18.7	21.6	24.2	25.8

^a^For physical activity and TV sitting time, quartiles were calculated from the ‘No Disease’ group so that their demarcators could be applied to disease group

^b^UK Government recommendations of 150mins of moderate or 75mins of vigorous activity per week. Walking was considered ‘moderate’ activity for this calculation

^c^Physiological thresholds used rather than quartiles because the shape of the risk relationship is a U shape (not linear like Physical activity and TV viewing)

**Fig. 3:**
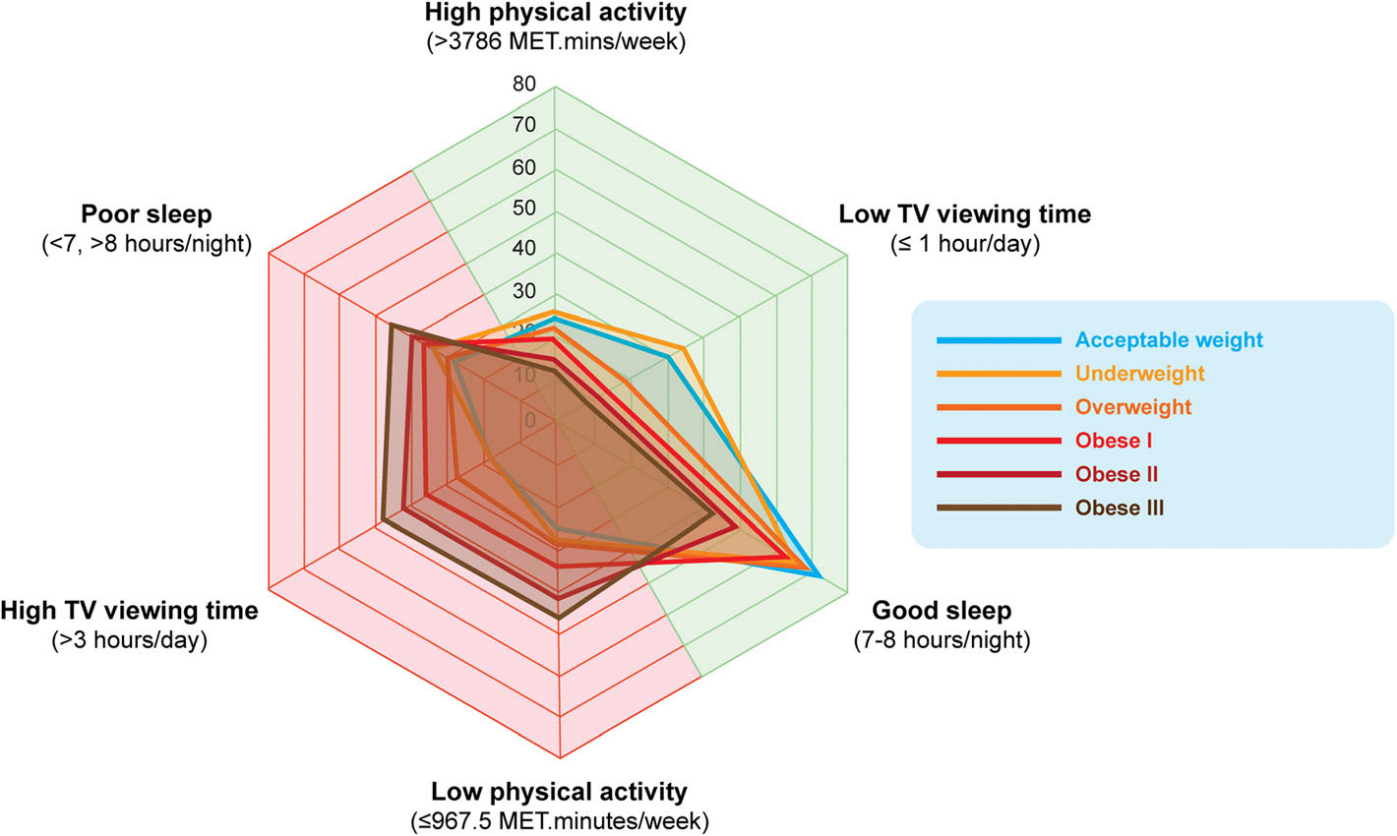
Radar chart showing the proportion of adults in each group who were categorised as either ‘high’ or ‘low’ for total physical activity or TV viewing, or ‘good’ or ‘poor’ for sleep duration. *Green side* indicates healthy lifestyle behaviours whereas *red side* indicates unhealthy behaviours. There is a shift leftwards towards unhealthy behaviours with an increase in BMI

Logistic regression models demonstrated that increased BMI is associated with a greater likelihood of reporting low physical activity, high TV viewing and poor sleep duration (Table 3). Indeed, ‘Obese III’ adults were 3 times more likely to report low physical activity (OR [95% CI] 3.13 [2.95–3.32]), 3 times more likely to report high TV viewing (3.26 [3.07–3.47]), and almost twice as likely to report poor sleep duration (1.78 [1.68–1.89]) than ‘Normal’ weight adults. The odds of reporting all three unhealthy behaviours together was higher than reporting one of these lifestyle behaviours individually. Relative to ‘Normal’ weight adults, ‘Obese III’ adults were 5 times (5.47 [4.96 to 6.05]) more likely to report an ‘unhealthy phenotype’ when controlling for confounders (Table 3). The shift in this unhealthy phenotype across BMI groups is visualised in Fig. 3 which shows the movement from healthy behaviours (green/right) to unhealthy behaviours (red/left).

**Table 3:** Odds [95% CI] of reporting unhealthy lifestyle behaviours separately and combined, across BMI groups

	Low physical activity	High TV viewing	Poor sleep	Low physical activity + High sitting + Poor sleep
<18.5 (underweight)	1.00 [0.86–1.16]	0.91 [0.77–1.07]	1.17 [1.02–1.35]	1.40 [0.96–2.02]
18.5–24.9 (normal weight)	1.00	1.00	1.00	1.00
25–29.9 (overweight)	1.23 [1.20–1.26]	1.52 [1.48–1.55]	1.09 [1.07–1.12]	1.44 [1.35–1.54]
30–34.9 (obese I)	1.66 [1.61–1.71]	2.06 [2.00–2.12]	1.31 [1.27–1.34]	2.38 [2.22–2.55]
35–39.9 (obese II)	2.21 [2.12–2.30]	2.69 [2.58–2.80]	1.50 [1.44–1.56]	3.49 [3.21–3.79]
>40 (obese III)	3.13 [2.95–3.32]	3.26 [3.07–3.47]	1.78 [1.68–1.89]	5.47 [4.96–6.05]

All Models adjusted for age, gender, socio-demographic (Townsend deprivation and ethnicity), smoking, alcohol, meets fruit + vegetable guidelines, cardio-metabolic disease and sleep apnoea

The odds of reporting unhealthy lifestyle behaviours increased according to waist circumference risk in the ‘Overweight’ and ‘Obese I’ groups. Furthermore, ‘Overweight’ individuals with a very high risk waist cm (Male >102 cm and Female >88 cm) were more likely to report low physical activity levels and the likelihood of reporting an ‘unhealthy phenotype’ was similar to ‘Obese’ adults with a low risk waist circumference (Male <94 and Female <80) (Additional file 1).

## Discussion

This is the largest UK population study to simultaneously assess physical activity, TV viewing and sleep duration across BMI groups. The results from the study indicate that these lifestyle behaviours cluster. Indeed obese adults are two to five times more likely to report an ‘unhealthy phenotype’, consisting of low physical activity, high TV viewing and poor sleep duration compared to normal weight adults. Physical activity, sedentary behaviour and poor sleep remain significant unaddressed risk factors in those who are overweight and obese.

Overweight and obese adults reported lower physical activity levels. Those with a BMI of ≥35, were two to three times more likely to report low levels of physical activity compared to normal weight adults. Global recommendations state adults should be performing at least 150 mins moderate or 75 mins vigorous physical activity weekly, but evidence states that this should be increased to 300 mins/week for additional health benefits [7, 32]. Data from the UK Biobank showed that almost 40% of adults with a BMI of ≥40 were not meeting the basic recommendations. A survey in England demonstrated that only 5% of adults could recall physical activity recommendations which was substantially lower than the 78% who could recall fruit and vegetable recommendations [33], highlighting a potential knowledge gap surrounding physical activity and health. There was a strong association between physical activity and waist cm, indeed overweight adults with a ‘very high risk waist cm’ had higher odds of reporting low physical activity, compared to obese I adults with a lower waist cm. These results are not surprising, considering that physical activity is strongly linked with visceral fat deposition. Although it’s clear that physical inactivity is a problem in obesity, a bi-directional relationship between physical activity and obesity is likely to exist [34].

TV viewing increased across BMI groups so that obese adults were 2–3 times more likely to report high levels compared to normal weight individuals. A number of cross sectional studies have demonstrated associations between sedentary behaviour and obesity, [17, 18, 35] however prospective data indicate bidirectional associations, whereby obesity leads to high sedentary time and vice versa [14, 15]. Unlike the strong associations between sedentary behaviour and cardio-metabolic risk, the relationship with obesity is less clear [36]. Whether high TV sitting time amongst obese individuals in the UK biobank is simply a proxy for low total daily energy expenditure, or whether it represents an independent risk for obesity remains to be determined, and more clinical trials are warranted. The most recent UK national guidelines for obesity briefly state that adults should limit TV viewing to 2 h per day [6] but results from this cohort indicate that more than half of overweight and obese adults exceed this limit on a daily basis. No further guidance on reducing sedentary behaviour is provided within current national and global guidelines. In 2014, the UK did produce the first ever UK strategy to embed physical activity and reduce sedentary behaviour into the fabric of daily life, [37] however, this was just a ‘framework for action’ rather than providing definite solutions to tackle sedentariness.

Sleep as a modifiable lifestyle behaviour in obesity prevention and management is rarely mentioned, despite data from this study showing a decline in good sleep (7–8 h per night) across BMI groups. Even when controlling for sleep apnoea, overweight and obese individuals were more likely to report short (<7 h) or long (>8 h) sleep duration. When sleep was measured objectively, a previous study also identified greater obesity risks with short sleep (<5 h) and demonstrated a ‘U’ shaped relationship with body fat [20]. Short sleep has been linked to metabolic dysregulation [38] and long sleep will clearly impact on the potential to be physically active during wakefulness. Nevertheless, the causal relationship between sleep and obesity have yet to be identified and a bidirectional relationship may exist, but these findings suggest sleep could be important in obesity pathogenesis.

These lifestyle behaviours seem to cluster, indeed the likelihood of reporting all three unhealthy behaviours (low physical activity, high TV viewing and poor sleep duration) was higher than reporting individual unhealthy behaviours in each BMI category. ‘Obese I’ adults were two times more likely and ‘Obese III’ were five times more likely to report an unhealthy behavioural phenotype compared to ‘normal weight ‘individuals. This indicates that the combination of unhealthy physical activity, TV viewing and sleep duration may expose individuals to greater obesity risk. It is possible that all three behaviours are interdependent on their influence upon energy balance and subsequent obesity risk. Muscular contraction during physical activity expends energy. Conversely, high sedentary behaviour decreases the daily work performed by large skeletal muscles in the back, legs and trunk large thereby reducing energy expenditure [12]. Energy intake has been linked to sleep through appetite control. Indeed sleep debt elevates ghrelin and reduces leptin which explains the strong link between sleep deprivation, raised energy intake and weight gain [13]. Collectively therefore, low physical activity, high sedentary behavior and poor sleep are likely to have a profound influence upon energy balance and weight gain. It is important to note that time spent in physical activity, sedentary behaviours and sleep are codependent, with a finite amount of time during the day, each behavior will influence the other [39].

This clustering of lifestyle behaviours suggests that to tackle the rising obesity trend, interventions solely targeting single lifestyle behaviours may be inadequate. To date, most intervention studies have focused on changing single lifestyle behaviours [22] but targeting multiple behaviours should be the focus of future strategies. The UK’s national clinical guidelines recently mentioned the possibility of lifestyle clustering and that unhealthy individuals may follow a range of unhealthy lifestyle behaviours which add to weight gain [6]. Our results confirm this hypothesis that overweight and obese individuals display a number of unhealthy behaviours which should all be prioritised in future interventions.

Dietary data indicate that overweight and obese UK adults were more likely to have changed their diet in the previous 5 years, consume a similar amount of fruit/vegetables, and less likely to eat sugar, compared to normal weight individuals. These behaviours are in line with existing guidelines [6, 8] however the results need to interpreted with caution. Self-report has major limitations in obese adults, [40] with significant underestimation of calorie intake. Although overweight and obese adults report making dietary changes, we cannot comment on the quality of these dietary changes. Over recent decades, food has become more varied, less expensive and more palatable, therefore dietary advice has been at the centre of lifestyle recommendations for tackling obesity. Despite the obvious limitations of this data, the results indicate that UK adults are at least aware of the importance of diet and emphasis should additionally be placed upon other lifestyle behaviours.

### Limitations

Strengths of the study are the large sample size allowing greater precision and resolution of associations, detailed measurements as well as the population based design. However, the response rate for the UK Biobank was only 5% which raises the strong possibility of selection bias and therefore reduced generalisability of results as this sample is unlikely to be a true representative of the general population. The cross sectional nature of this study means we cannot comment on the direction of causality. Additionally, lifestyle behaviours were self-report which is known to lead to over-reporting with physical activity data [41]. That being said, all questionnaires used in the study were validated and more easily adopted for population wide studies compared to objective measures. Using TV viewing as a proxy measure of sedentary behaviour does not take into account the fact that TV viewing and weight outcomes may be caused by several factors such as snacking during TV, and being prompted to eat by TV. Nonetheless, TV sitting time is a commonly used measure of sedentary behaviour in epidemiological studies and has good test-retest reliability [42]. Calculation of total daily sitting time, which is a more complete measure, was not possible from the UK Biobank questionnaire used.

## Conclusions

In summary, the present data indicate that overweight and obese adults report low levels of physical activity, high TV viewing and poor sleep duration. These lifestyle behaviours seem to cluster and may collectively expose individuals to greater risk of obesity. Those who are obese are two to five times more likely to display an ‘unhealthy phenotype’ compared to normal weight adults. Current guidelines for obesity recommend pharmacological intervention to begin only once lifestyle have been implemented. Despite this emphasis on lifestyle, the results from the UK Biobank suggest that physical activity, sedentary behaviour and sleep remain significant unaddressed risk factors for obesity. This highlights the need for more effective strategies, which target multiple lifestyle behaviours, to prevent the rising tide of obesity.

## Additional file

Additional file 1: The socio-demographics of the missing cases and logistic regression analysis with waist circumference. (DOCX 27 kb)

## Abbreviations

BMI: Body Mass Index
IPAQ: International Physical Activity Questionnaire
NICE: National Institute for Health and Care Excellence
TV: Television
WHO: World Health Organisation
