# Supplementary material for: Low physical activity, high television viewing and poor sleep duration cluster in overweight and obese adults; a cross-sectional study of 398,984 participants from the UK Biobank
Source: Int J Behav Nutr Phys Act. 2017 Apr 28;14:57. doi: 10.1186/s12966-017-0514-y (PMC5408822; doi:10.1186/s12966-017-0514-y)
Supplement: Additional file 1: — The socio-demographics of the missing cases and logistic regression analysis with waist circumference. (DOCX 27 kb) [file 12966_2017_514_MOESM1_ESM.docx]

**Additional material**

**Table 1 Socio-demographics of those who have missing data on physical activity, sitting or sleep and therefore excluded from analysis (n=100,574).**

|  | **% within each disease group** | | | | | |
| --- | --- | --- | --- | --- | --- | --- |
|  | **Normal weight**  **18.5-24.99**  ***(n=30,164)*** | **Under**  **weight**  ***<18.5***  ***(n=600*)** | **Over**  **weight**  **25-29.99**  ***(n=41,137)*** | **Obese I**  **30-34.99**  ***(n=19,630)*** | **Obese II 35-39.99 *(n=6344)*** | **Obese III ≥40 *(n=2699)*** |
| **SOCIO-DEMOGRAPHICS** | | | | | | |
| **% Male** | 28.9 | 20.5 | 43.7 | 41.2 | 31.4 | 23.9 |
| **Age *(n)*** | *30,164* | *600* | *41,137* | *19,630* | *6344* | *2699* |
| 40-49 | 23.3 | 25.2 | 18.6 | 17.3 | 17.7 | 22.0 |
| 50-59 | 33.8 | 37.3 | 31.4 | 32.1 | 34.8 | 37.0 |
| 60-70 | 42.9 | 37.5 | 49.9 | 50.6 | 47.6 | 41.0 |
| **Waist cm groups (MALES) *(n)*** | *8709* | *123* | *17,954* | *8083* | *1993* | *644* |
| <94 (low risk) | 88.3 | 100.0 | 33.2 | 1.8 | 0.1 | 0.0 |
| 94-102 (high risk) | 11.3 | 0 | 50.8 | 22.6 | 1.4 | 0.0 |
| >102 (very high risk) | 0.3 | 0 | 16.1 | 75.6 | 98.5 | 100.0 |
| **Waist cm groups (FEMALES) *(n)*** | *21,450* | *477* | *23,173* | *11,541* | *4346* | *2053* |
| <80 (low risk) | 76.0 | 98.1 | 17.8 | 0.7 | 0.0 | 0.0 |
| 80-88 (high risk) | 21.7 | 1.9 | 48.4 | 12.9 | 1.1 | 0.0 |
| >88 (very high risk) | 2.4 | 0.0 | 33.9 | 86.4 | 98.9 | 100% |
| **Cardio-metabolic disease** | 43.2 | 36.8 | 60.3 | 75.9 | 85.0 | 89.6 |
| **Sleep apnoea (n)** | 0.0 (15) | 0.0 (0) | 0.2 (81) | 0.5 (103) | 1.0 (63) | 2.3 (61) |
| **Townsend deprivation quintile *(n)*** | *30,131* | *598* | *41,094* | *19,610* | *6336* | *2696* |
| 1 (least deprived) | 20.2 | 17.6 | 18.9 | 15.5 | 13.3 | 10.2 |
| 2 | 19.8 | 16.9 | 19.0 | 17.2 | 15.2 | 13.3 |
| 3 | 19.7 | 15.1 | 20.0 | 19.3 | 17.1 | 16.2 |
| 4 | 19.4 | 19.2 | 20.0 | 20.2 | 22.2 | 21.4 |
| 5 (most deprived) | 20.9 | 31.3 | 22.1 | 27.8 | 32.4 | 38.9 |
| **Ethnicity *(n)*** | *29,846* | *588* | *40,643* | *19,374* | *6266* | *2664* |
| White/British | 94.2 | 94.0 | 93.4 | 93.2 | 92.5 | 91.9 |
| Mixed | 0.6 | 0.5 | 0.5 | 0.6 | 0.7 | 0.8 |
| Asian | 2.2 | 2.0 | 2.7 | 2.1 | 2.2 | 1.6 |
| Black African | 1.3 | 0.3 | 1.9 | 2.7 | 3.4 | 4.4 |
| Chinese | 0.7 | 1.9 | 0.2 | 0.1 | 0.0 | 0.0 |
| Other | 0.9 | 1.2 | 1.2 | 1.2 | 1.2 | 1.3 |
| **Smoking (n)** | *30,021* | *595* | *40,931* | *19,513* | *6307* | *2693* |
| Never | 57.5 | 52.8 | 53.4 | 51.0 | 52.5 | 53.9 |
| Previous | 27.8 | 19.7 | 34.2 | 37.3 | 36.7 | 35.9 |
| Current | 13.9 | 26.9 | 11.4 | 10.5 | 9.6 | 9.2 |
| Prefer not to answer | 0.8 | 0.7 | 0.9 | 1.1 | 1.2 | 1.0 |
| **Alcohol (n)** | *30,021* | *595* | *40,931* | *19,512* | *6307* | *2683* |
| Never | 5.6 | 9.7 | 5.6 | 6.7 | 8.5 | 10.8 |
| Previous | 3.7 | 9.1 | 3.7 | 4.7 | 6.4 | 8.4 |
| Current | 90.4 | 80.2 | 90.4 | 88.2 | 84.6 | 80.5 |
| Prefer not to answer | 0.3 | 1.0 | 0.4 | 0.4 | 0.5 | 0.3 |

**Table 2 Odds [95% CI] of reporting unhealthy lifestyle behaviours separately and combined, across BMI and waist circumference groups.** *Due to the spread of waist circumference in ‘overweight’ and ‘obese I’ groups, we classified only these groups by waist circumference.* *All* *Models adjusted for age, gender, socio-demographic (Townsend deprivation and ethnicity), smoking, alcohol, diet, cardio-metabolic disease and sleep apnoea.*

|  | **Low**  **physical**  **activity** | **High TV viewing** | **Poor**  **sleep** | **Low Physical Activity +**  **High Sitting**  **+**  **Poor Sleep** |
| --- | --- | --- | --- | --- |
| **Normal weight** | 1.00 | 1.00 | 1.00 | 1.00 |
| **Underweight** | 0.98 [0.85-1.14] | 0.90 [0.76-1.07] | 1.17 [1.02-1.35] | 1.38 [0.95-2.01] |
| **Overweight**  ***‘low risk waist circumference’*** | 1.09 [1.06-1.12] | 1.44 [1.41-1.49] | 1.07 [1.05-1.10] | 1.28 [1.19-1.38] |
| **Overweight**  ***‘high risk waist circumference’*** | 1.42 [1.37-1.46] | 1.60 [1.55-1.65] | 1.12 [1.09-1.15] | 1.59 [1.46-1.72] |
| **Overweight**  ***‘very high risk waist circumference’*** | 1.95 [1.85-2.04] | 1.81 [1.72-1.90] | 1.14 [1.09-1.20] | 2.13 [1.91-2.39] |
| **Obese 1**  ***‘low risk waist circumference’*** | 1.48 [1.40-1.56] | 1.90 [1.80-2.01] | 1.28 [1.21-1.35] | 2.14 [1.88-2.42] |
| **Obese 1**  ***‘high risk waist circumference’*** | 1.48 [1.42-1.54] | 2.03 [1.95-2.11] | 1.28 [1.23-1.33] | 2.04 [1.86-2.25] |
| **Obese 1**  ***‘very high risk waist circumference’*** | 1.96 [1.90-2.03] | 2.18 [2.10-2.26] | 1.34 [1.29-1.38] | 2.78 [2.56-3.01] |
| **Obese 11** | 2.25 [2.16-2.35] | 2.71 [2.60-2.82] | 1.50 [1.44-1.56] | 3.53 [3.25-3.84] |
| **Obese 111** | 3.16 [2.98-3.36] | 3.3 [3.08-3.48] | 1.78 [1.68-1.89] | 5.49 [4.97-6.06] |
